# Supplementary material for: Regulating the electronic structure through charge redistribution in dense single-atom catalysts for enhanced alkene epoxidation
Source: Nat Commun. 2023 Apr 29;14:2494. doi: 10.1038/s41467-023-38310-1 (PMC10148878; doi:10.1038/s41467-023-38310-1)
Supplement: Supplementary file 1 — Supplementary Information [file 41467_2023_38310_MOESM1_ESM.pdf]

Supplementary Information

Regulating the electronic structure through charge redistribution in dense single-atom catalysts for enhanced alkene epoxidation

Hongqiang Jin,[1, 2#] Kaixin Zhou,[1, 2#] Ruoxi Zhang,[1, 2] Hongjie Cui,[1] Yu Yu,[3✉] Peixin Cui,[4✉] Weiguo Song[1, 2] and Changyan Cao[1, 2✉]

[1]Beijing National Laboratory for Molecular Sciences, CAS Research/Education Center for Excellence in Molecular Sciences, Laboratory of Molecular Nanostructures and Nanotechnology, Institute of Chemistry, Chinese Academy of Sciences, Beijing 100190, PR China. [2]School of Chemical Sciences, University of Chinese Academy of Sciences, Beijing 100049, PR China. [3]Department of Materials Science and Engineering, Beijing Jiaotong University, Beijing 100044, PR China. [4]Key Laboratory of Soil Environment and Pollution Remediation, Institute of Soil Science, Chinese Academy of Sciences, Nanjing 210008, PR China. [#]These authors contributed equally.
✉E-mail: yuyu@bjtu.edu.cn; pxcui@issas.ac.cn; cycao@iccas.ac.cn

Supplementary Figures and Tables

Supplementary Fig. 1 Schematic illustration the synthesis procedures of M-SACs.

Supplementary Fig. 2 SEM images of as-prepared two-dimensional nanosheets.

Supplementary Fig. 3 XRD patterns of various M_1/NOC SACs samples.

Supplementary Fig. 4 Characterizations of Fe_1/NOC.

Supplementary Fig. 5 Characterizations of Ni_1/NOC.

Supplementary Fig. 6 Characterizations of Cu_1/NOC.

Supplementary Fig. 7 Characterizations of Zn_1/NOC.

Supplementary Fig. 8 Characterizations of Ru_1/NOC.

Supplementary Fig. 9 Characterizations of Ir_1/NOC.

Supplementary Fig. 10 Atomic structural analysis of M-SACs.

Supplementary Fig. 11 N 1s XPS spectrum of various M-SACs samples.

Supplementary Fig. 12 O 1s XPS spectrum of various M-SACs samples.

Supplementary Fig. 13 SEM images of Co_1/NOC-x SACs samples.

Supplementary Fig. 14 XRD patterns of various Co_1/NOC-x samples.

Supplementary Fig. 15 Characterizations of Co_1/NOC-5.

Supplementary Fig. 16 Characterizations of Co_1/NOC-11.

Supplementary Fig. 17 Characterizations of Co_1/NOC-21.

Supplementary Fig. 18 Co 2p XPS spectrum of various Co_1/NOC-x samples.

Supplementary Fig. 19 k^3-weight FT-EXAFS spectra of Co_1/NOC-21.

Supplementary Fig. 20 Raman spectra and C *1s* XPS spectra of Co_1/NOC-x samples.

Supplementary Fig. 21 The structure models of various Co_1/NOC-x samples and the corresponding theoretical Co loadings.

Supplementary Fig. 22 Formation energies of Co_1-N_3O_1 configuration models with varying Co density by DFT calculations.

Supplementary Fig. 23 The corresponding spin moment originated from the spin-splitting of Co 3d atom orbitals in x-Co_1-N_3O_1 models.

Supplementary Fig. 24 SB conversion against reaction time and Arrhenius plots as well as corresponding apparent activation energies of the Co_1/NOC-x catalysts.P

Supplementary Fig. 25 Recycling performances in trans-stilbene epoxidation with Co_1/NOC-21 sample.

Supplementary Fig. 26 Characterizations of Co_1/NOC-used.

Supplementary Fig. 27 Atomic structural analysis of Co_1/NOC-used.

Supplementary Fig. 28 Atomic structural analysis of gram-scale Co SAC.

Supplementary Fig. 29 ^{1}H NMR spectra of generated SBO.

Supplementary Fig. 30 ^{13}C NMR spectra of generated SBO.
Supplementary Fig. 31 O-O bond after adsorption on Co atom and vacancy sites.
Supplementary Fig. 32 Scheme of the energy levels of Co and O and charge transfer.
Supplementary Fig. 33 The spin density isosurfaces of various x-Co_1-N_3O_1 models.
Supplementary Fig. 34 The corresponding spin moment on x-Co_1-N_3O_1 models.
Supplementary Fig. 35 Top view of the intermediate configurations of 4-Co_1-N_3O_1.
Supplementary Fig. 36 The reaction pathways and the configurations of intermediates over 1-Co_1-N_3O_1.
Supplementary Fig. 37 The reaction pathways and the configurations of intermediates over 2-Co_1-N_3O_1.
Supplementary Fig. 38 The reaction pathways for formation of epoxy benzene and phenol.
Supplementary Fig. 39 XRD pattern of Co NPs/NC sample.
Supplementary Fig. 40 Characterizations of Co NPs/NC sample.
Supplementary Fig. 41 Energy profiles of trans-stilbene epoxidation reaction on Co(111) model.
Supplementary Table 1. ICP results of various M_1/NOC samples.
Supplementary Table 2. EXAFS fitting parameters for various M-SACs samples.
Supplementary Table 3. ICP results of various Co_1/NOC-x samples.
Supplementary Table 4. EXAFS fitting parameters at the Co K-edge
Supplementary Table 5. XPS results of various Co_1/NOC-x samples.
Supplementary Table 6. Bader charge of various x-Co_1-N_3O_1 models.
Supplementary Table 7. The spin moments of Co atoms in different x-Co_1-N_3O_1 models.
Supplementary Table 8. Substrate scope of alkene epoxidation over the Co_1/NOC-21 catalyst.
Supplementary Table 9. The spin moments of Co atoms in different O_2-adsorbed x-Co_1-N_3O_1 models.
Supplementary Table 10. The spin moments of O atoms in different x-Co_1-N_3O_1 models before and after adsorption.
Supplementary Table 11. The sole value of imaginary frequencies of TS-1 and TS-2 on various x-Co_1-N_3O_1 models.
Supplementary Table 12. Free energies of the reaction intermediates with different Co_1-N_3O_1 models.

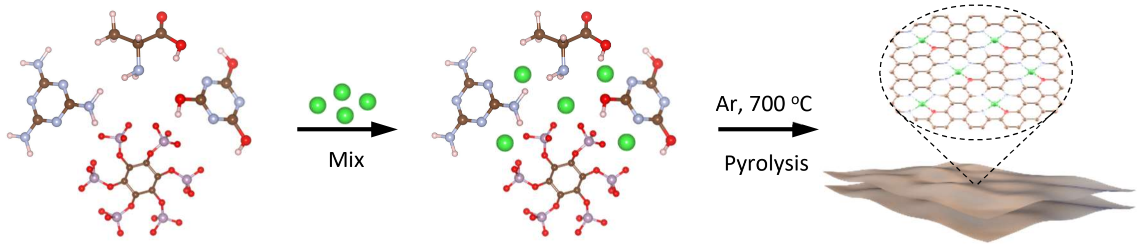

Supplementary Fig. 1 Schematic illustration the synthesis procedures of M-SACs.

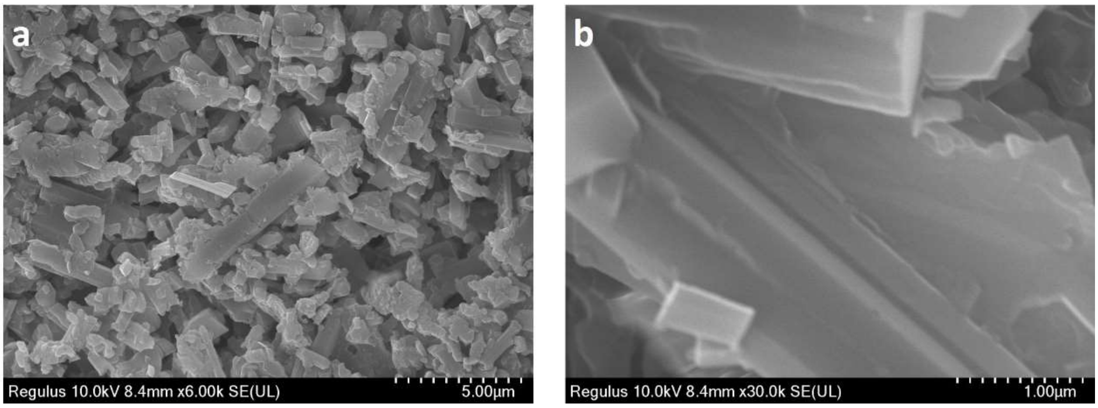

Supplementary Fig. 2 SEM images of as-prepared two-dimensional nanosheets under (a) low magnification and (b) high magnification.

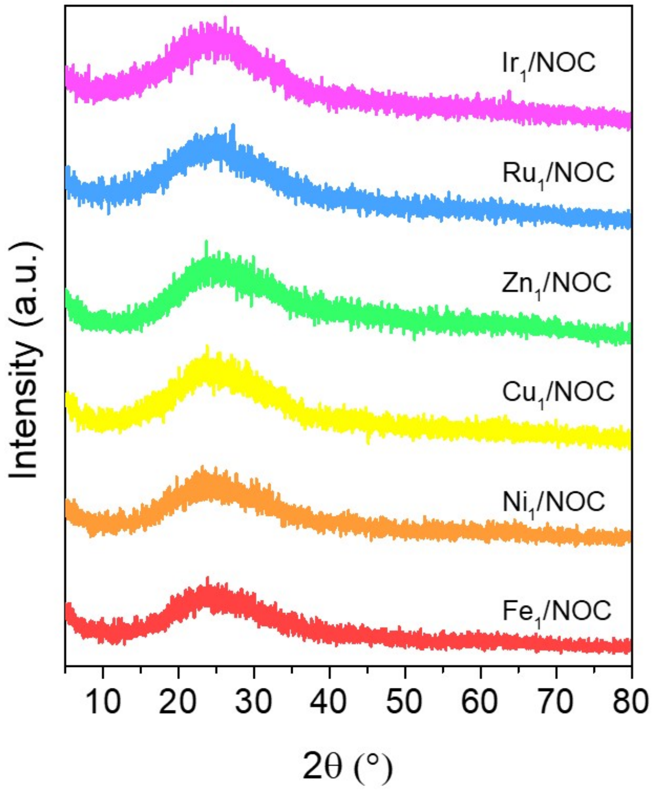

Supplementary Fig. 3 XRD patterns of various M_1/NOC SACs samples.

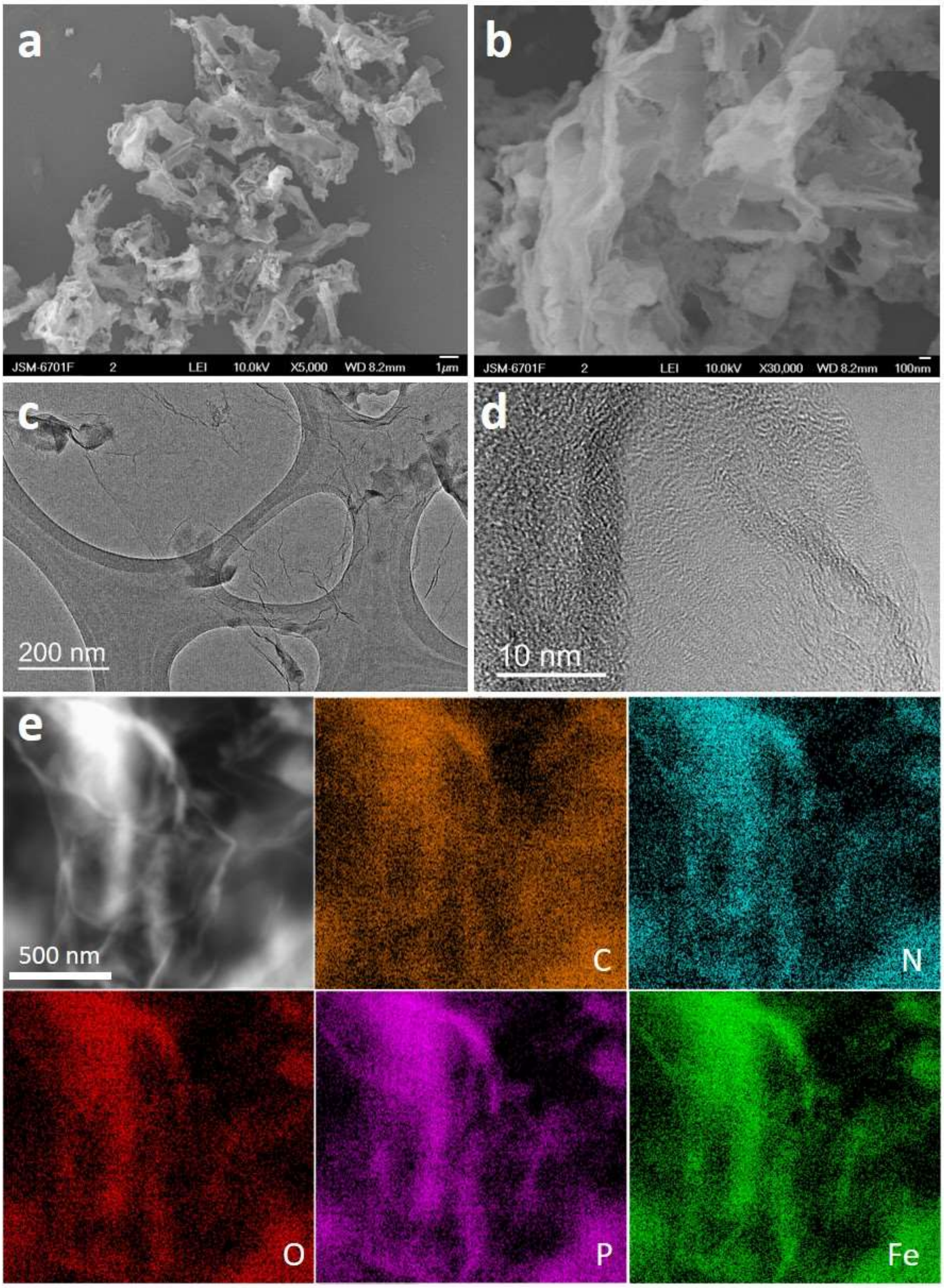

Supplementary Fig. 4 Characterizations of Fe_1/NOC. (a-b) SEM images of Fe_1/NOC. (c) TEM and (d) HRTEM images of Fe_1/NOC. (e) HAADF images and corresponding EDS mapping of Fe_1/NOC.

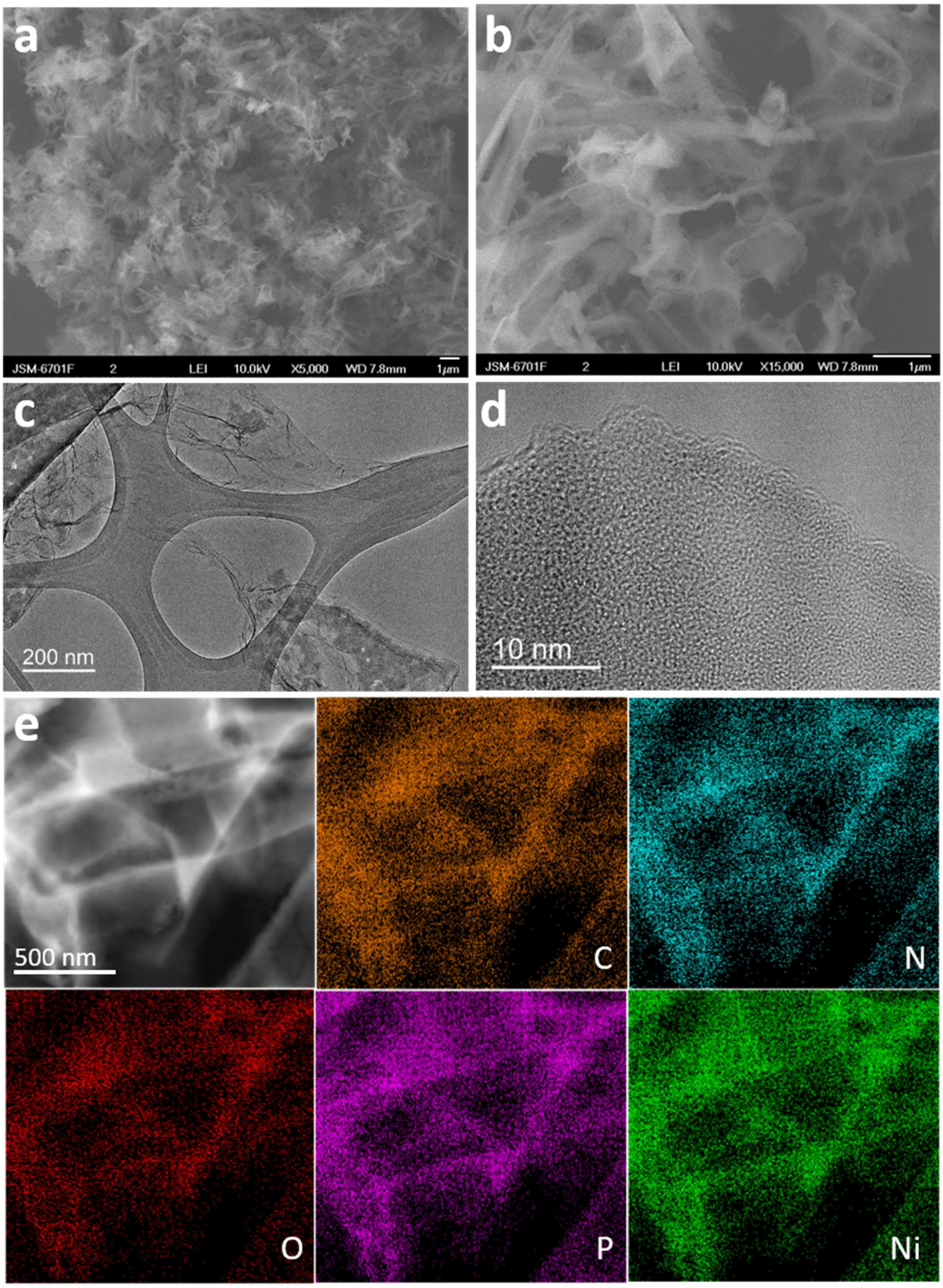

Supplementary Fig. 5 Characterizations of Ni_1/NOC. (a-b) SEM images of Ni_1/NOC. (c) TEM and (d) HRTEM images of Ni_1/NOC. (e) HAADF images and corresponding EDS mapping of Ni_1/NOC.

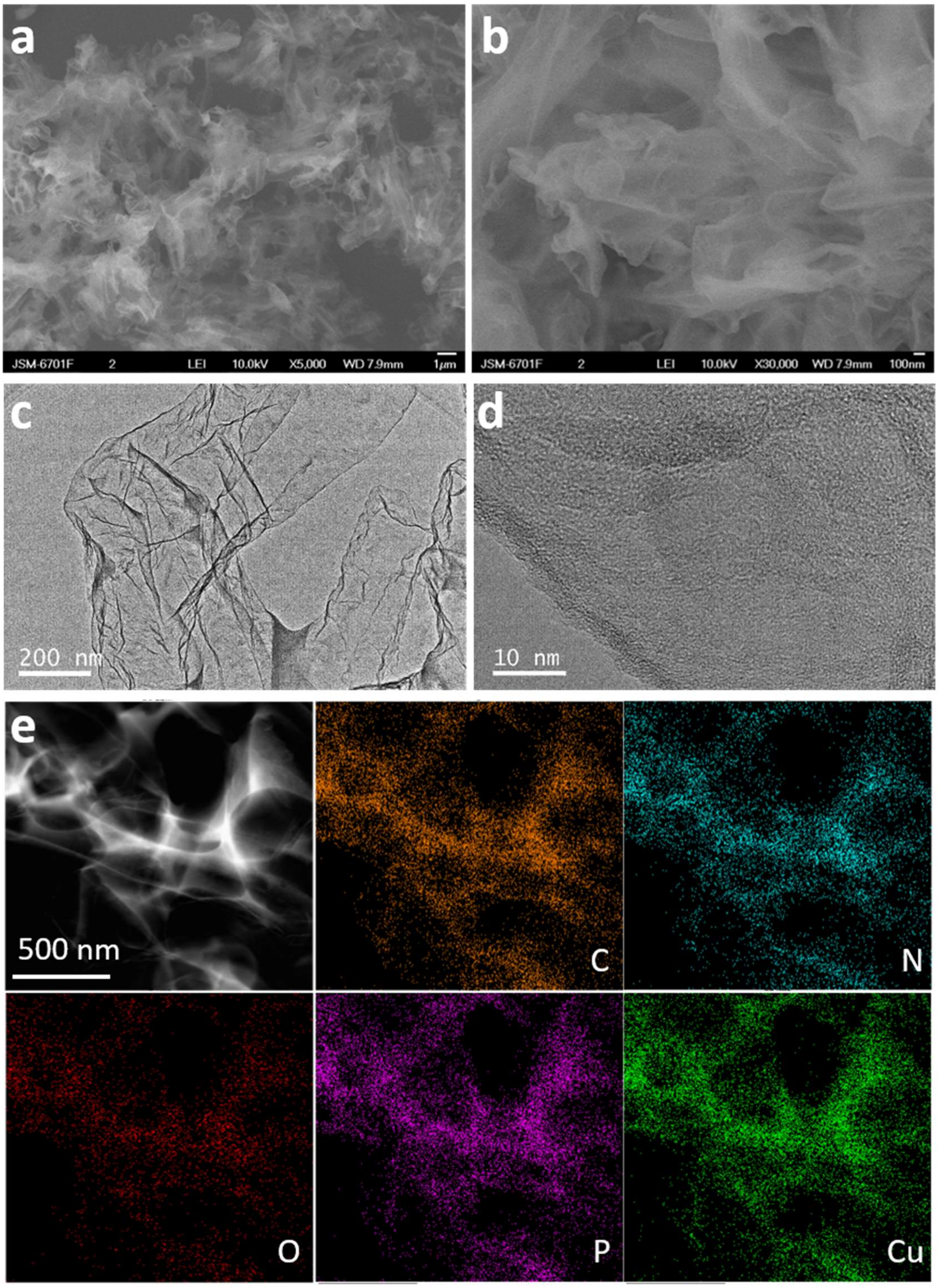

Supplementary Fig. 6 Characterizations of Cu_1/NOC. (a-b) SEM images of Cu_1/NOC. (c) TEM and (d) HRTEM images of Cu_1/NOC. (e) HAADF images and corresponding EDS mapping of Cu_1/NOC.

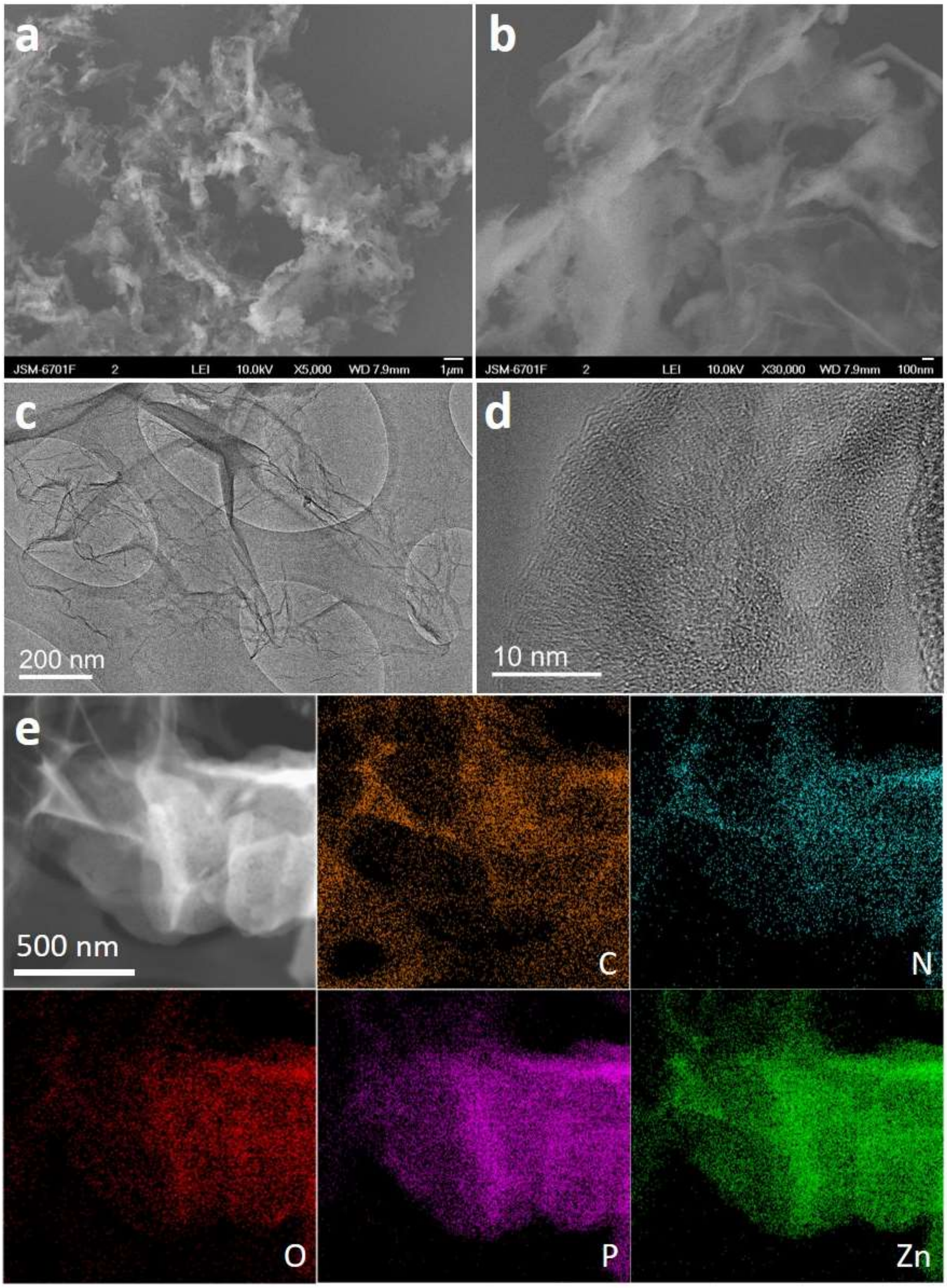

Supplementary Fig. 7 Characterizations of Zn_1/NOC. (a-b) SEM images of Zn_1/NOC. (c) TEM and (d) HRTEM images of Zn_1/NOC. (e) HAADF images and corresponding EDS mapping of Zn_1/NOC.

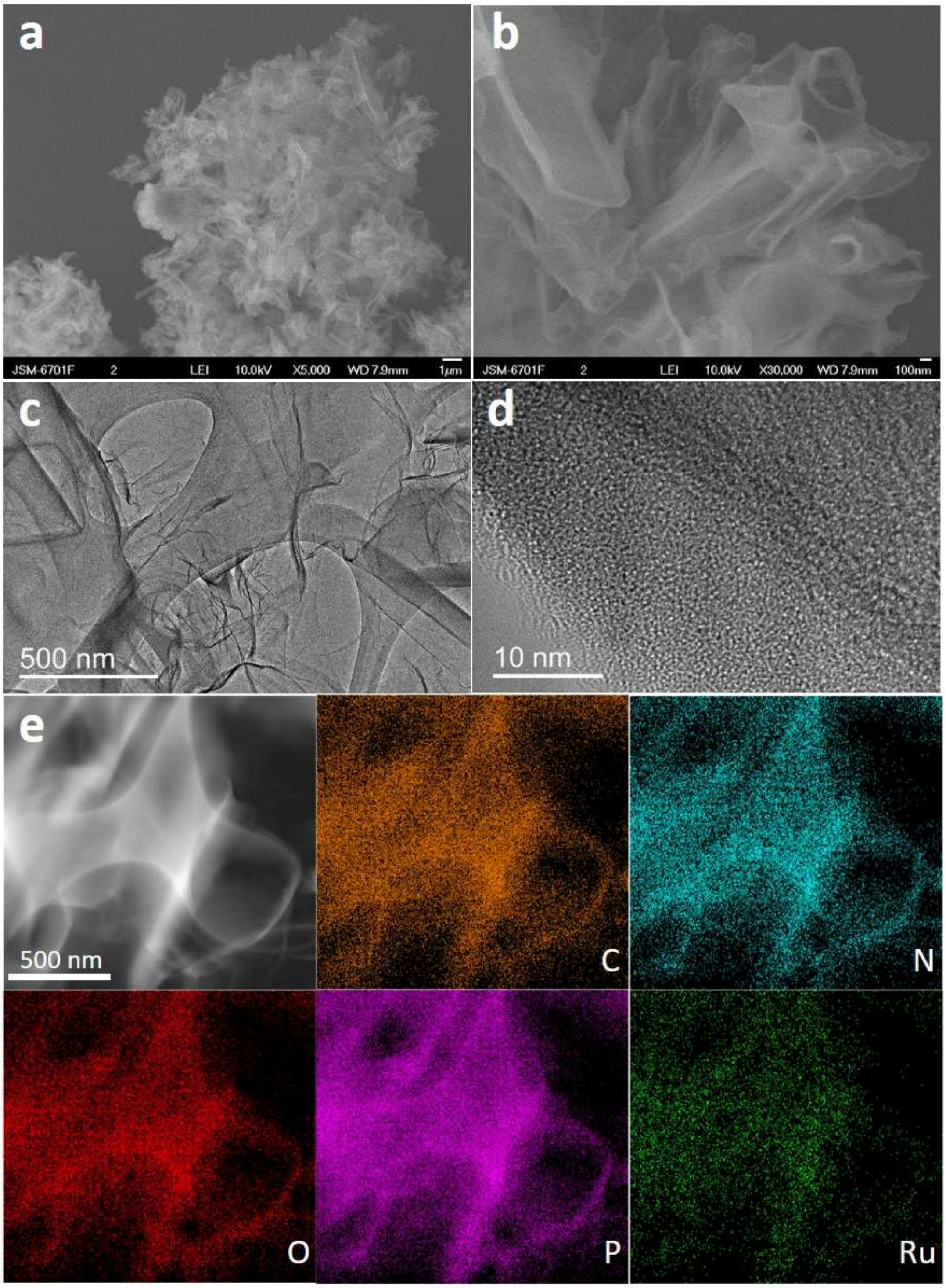

Supplementary Fig. 8 Characterizations of Ru_1/NOC. (a-b) SEM images of Ru_1/NOC. (c) TEM and (d) HRTEM images of Ru_1/NOC. (e) HAADF images and corresponding EDS mapping of Ru_1/NOC.

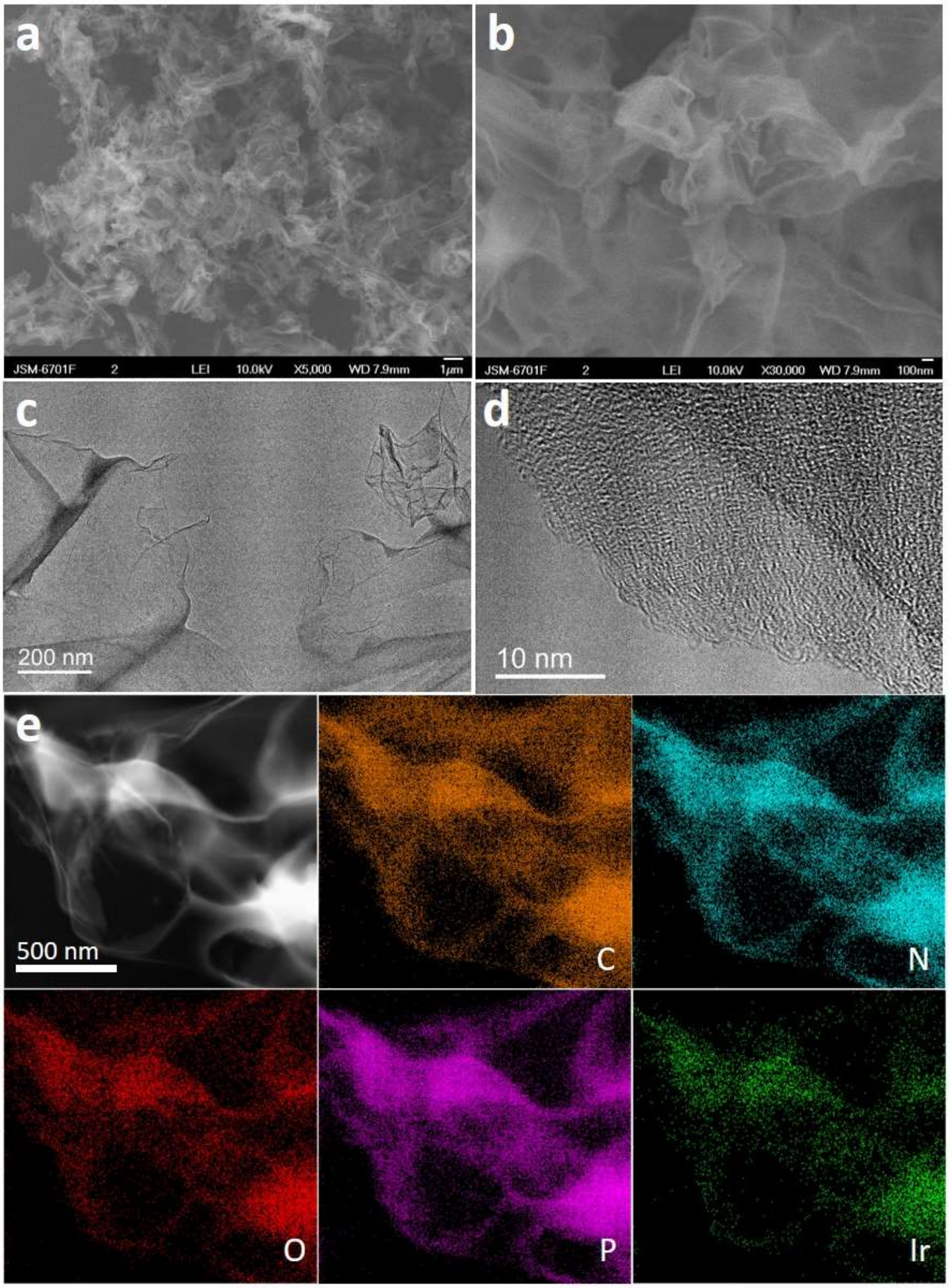

Supplementary Fig. 9 Characterizations of Ir_1/NOC. (a-b) SEM images of Ir_1/NOC. (c) TEM and (d) HRTEM images of Ir_1/NOC. (e) HAADF images and corresponding EDS mapping of Ir_1/NOC.

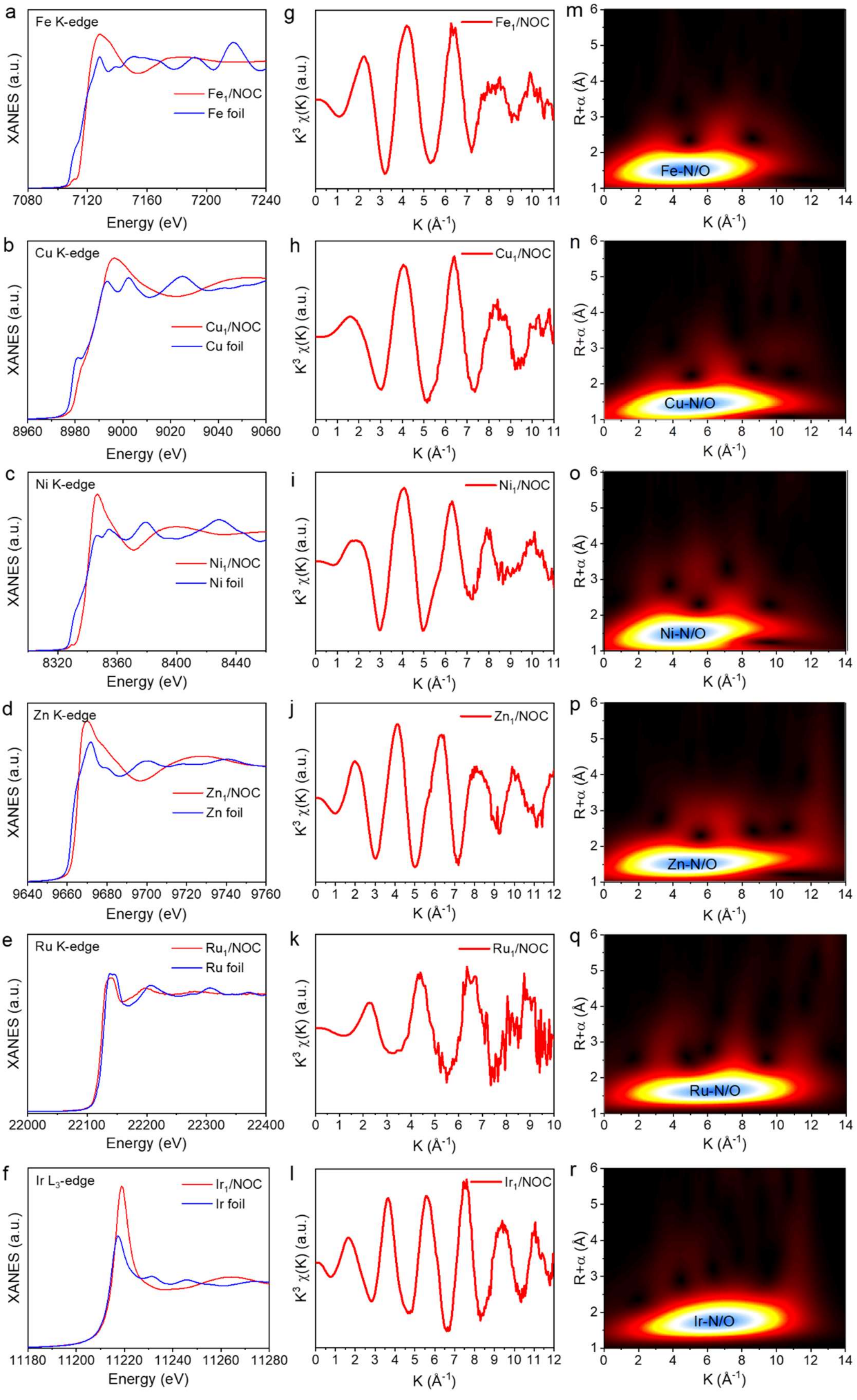

Supplementary Fig. 10 Atomic structural analysis of M-SACs. (a-f) Normalized XANES spectra of various M-SACs samples. (g-l) k space of various M-SACs samples. (m-r) Wavelet transform

(WT) analysis.

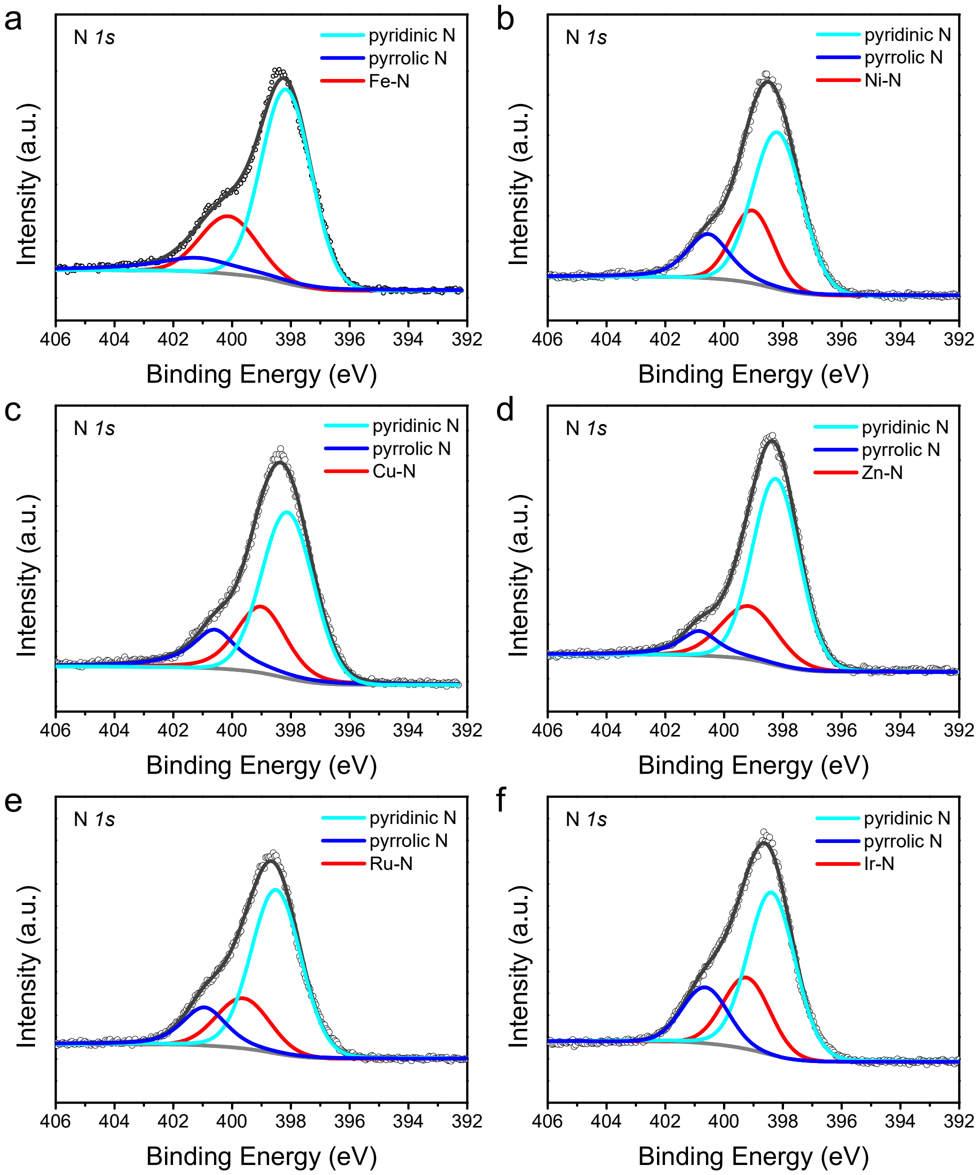

Supplementary Fig. 11 N 1s XPS spectrum of various M-SACs samples. (a) Fe_1/NOC, (b) Ni_1/NOC, (c) Cu_1/NOC, (d) Zn_1/NOC, (e) Ru_1/NOC, and (f) Ir_1/NOC.

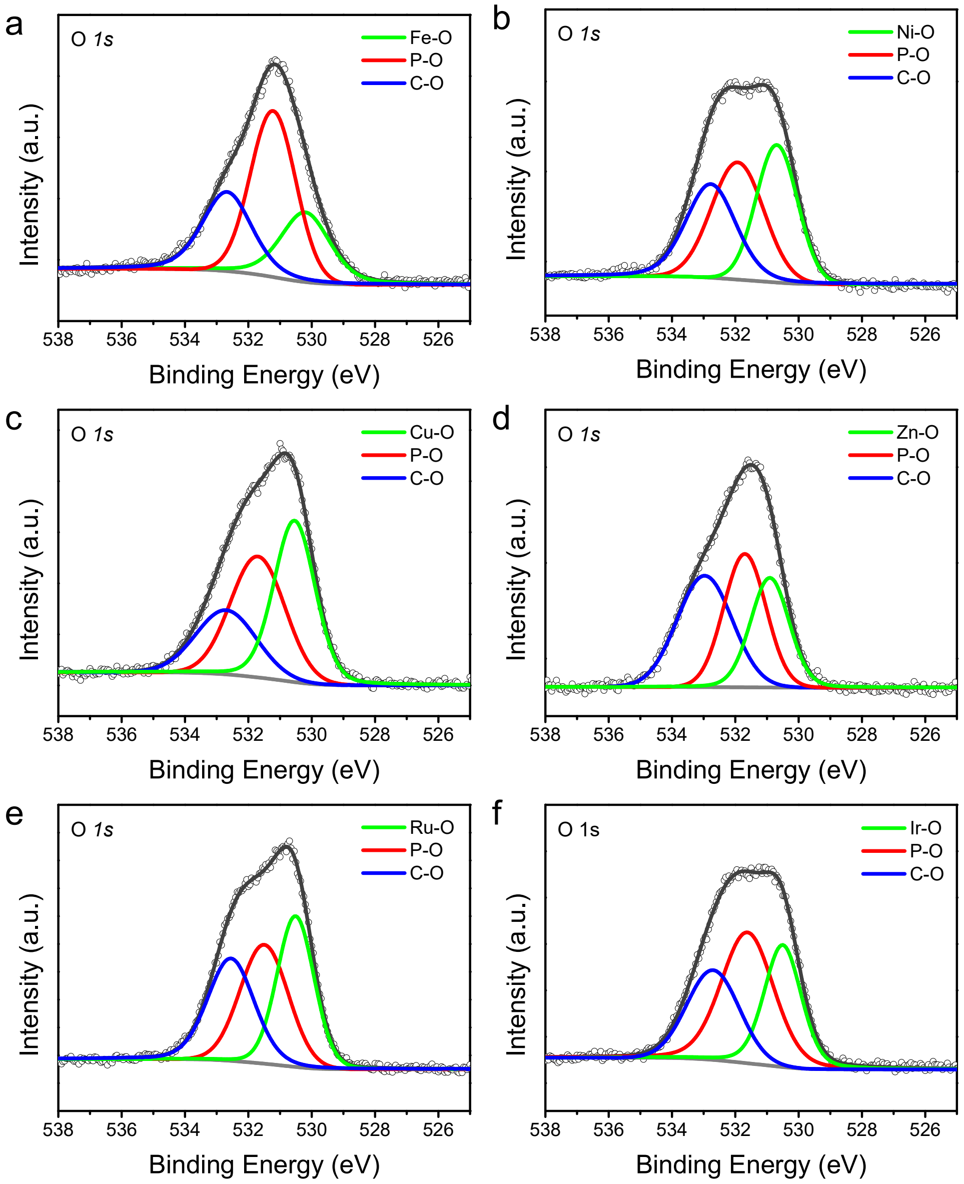

Supplementary Fig. 12 O 1s XPS spectrum of various M-SACs samples. (a) Fe_1/NOC, (b) Ni_1/NOC, (c) Cu_1/NOC, (d) Zn_1/NOC, (e) Ru_1/NOC, and (f) Ir_1/NOC.

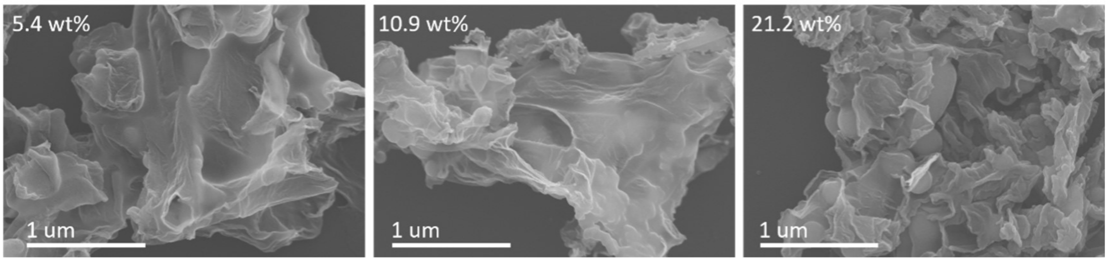

Supplementary Fig. 13 SEM images of 5.4 wt%, 10.9 wt%, and 21.2 wt% Co SACs samples.

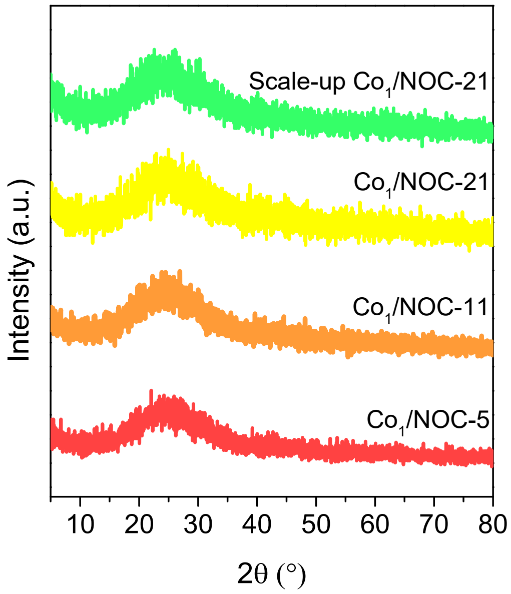

Supplementary Fig. 14 XRD patterns of various Co_1/NOC-x samples.

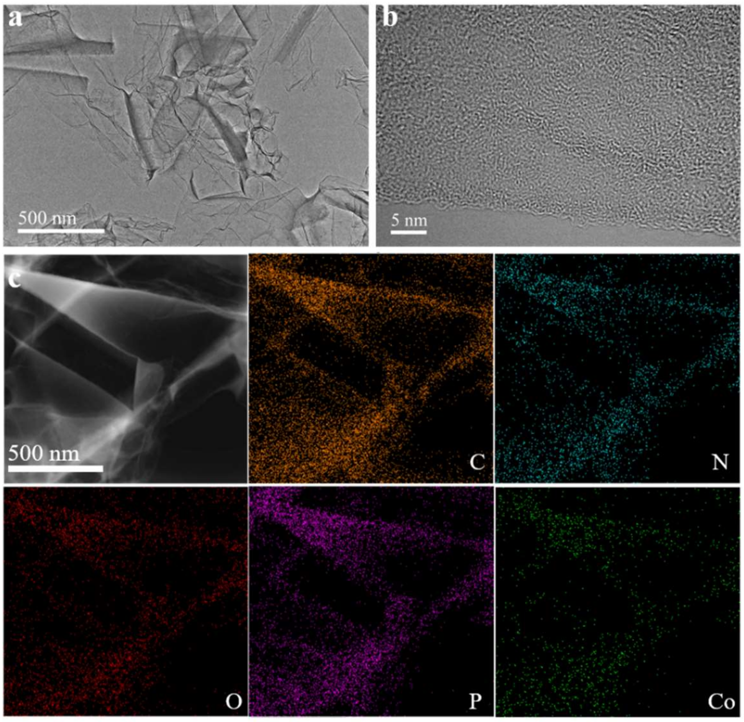

Supplementary Fig. 15 Characterizations of Co_1/NOC-5. (a) TEM and (b) HRTEM images of Co_1/NOC-5. (c) HAADF images and corresponding EDS mapping of Co_1/NOC-5.

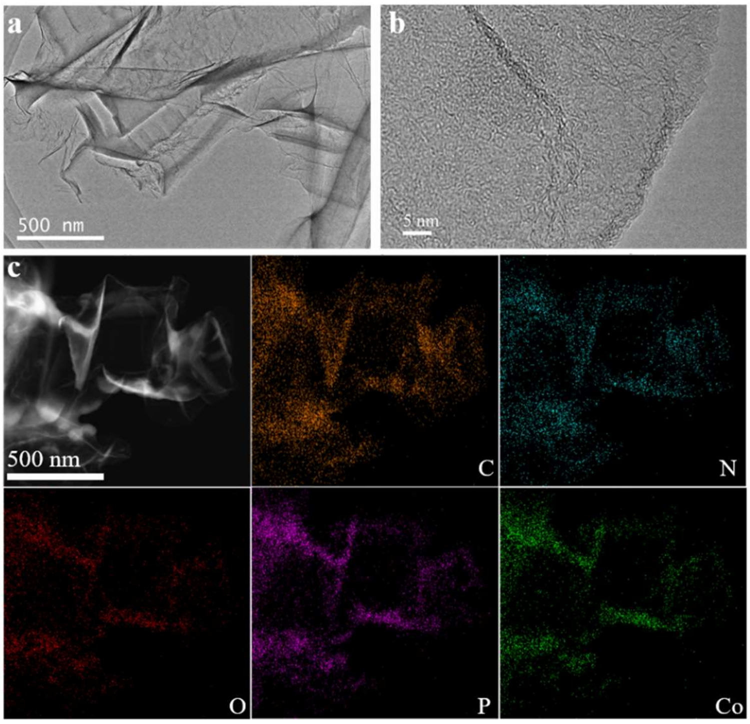

Supplementary Fig. 16 Characterizations of Co_1/NOC-11. (a) TEM and (b) HRTEM images of Co_1/NOC-11. (c) HAADF images and corresponding EDS mapping of Co_1/NOC-11.

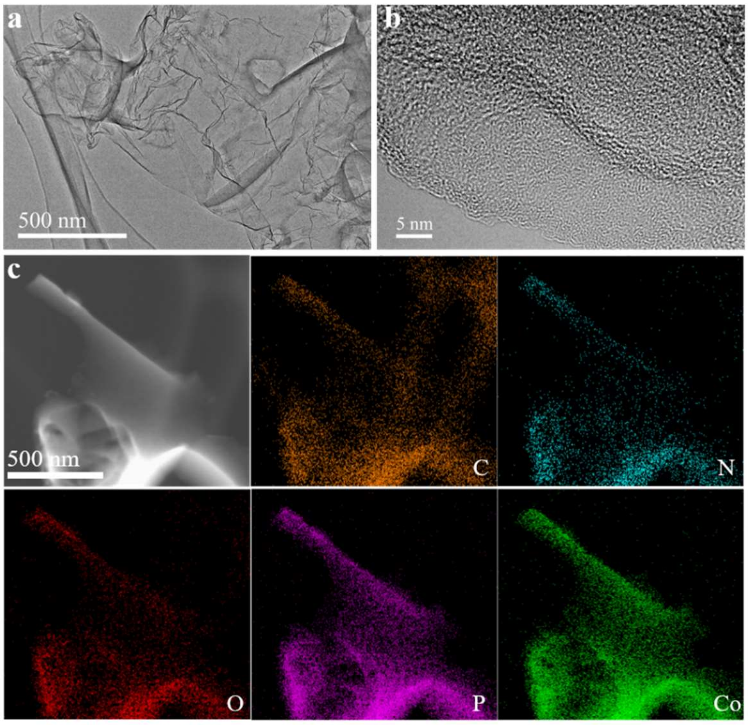

Supplementary Fig. 17 Characterizations of Co_1/NOC-21. (a) TEM and (b) HRTEM images of Co_1/NOC-21. (c) HAADF images and corresponding EDS mapping of Co_1/NOC-21.

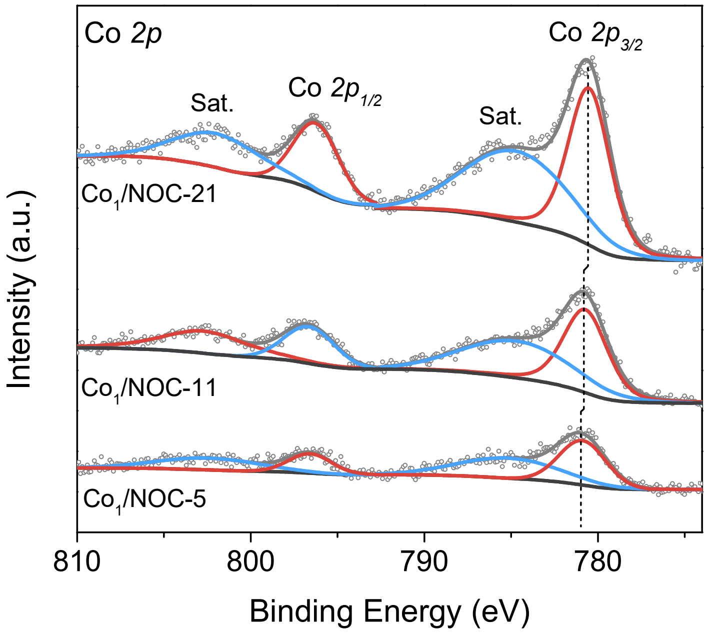

Supplementary Fig. 18 Co 2p XPS spectrum of various Co_1/NOC-x samples.

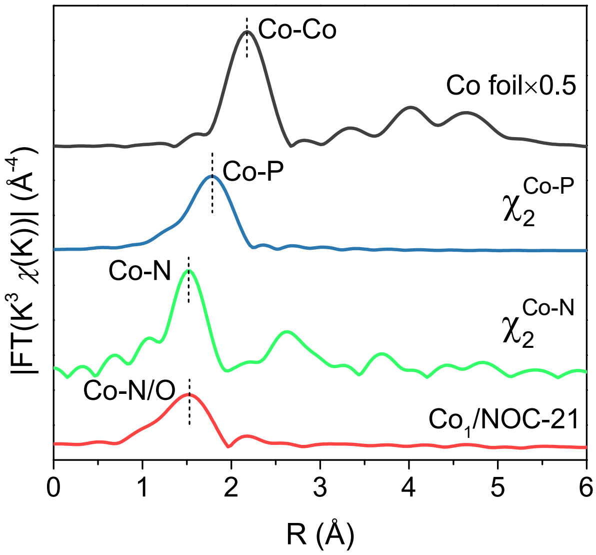

Supplementary Fig. 19 k^3-weight FT-EXAFS spectra of Co_1/NOC-21. Curves from top to bottom are the Co-P, Co-N backscattering pathways.

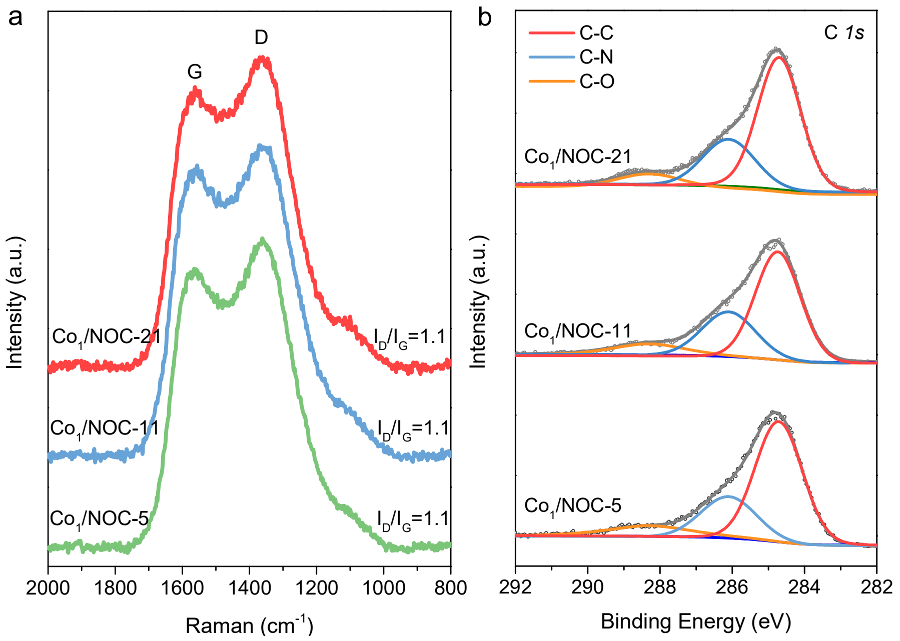

Supplementary Fig. 20 (a) Raman spectra and (b) C *1s* XPS spectra of Co_1/NOC-x samples.

a

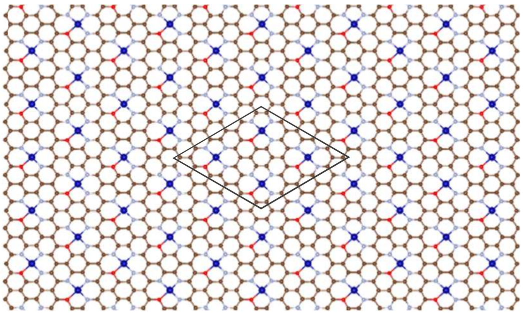

Sample: Co_1/NOC-21

Unit cell: 48C, 12N, 4O, 4Co

Theoretical Co loading:

$$\text{Co (wt\%)} = \frac{4\times59}{48\times12+\quad\times14+4\times16+4\times59}$$

$$\approx 22\ \text{wt\%}$$

b

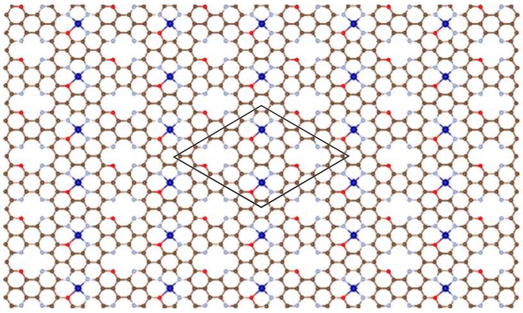

Sample: Co_1/NOC-11

Unit cell: 48C, 12N, 4O, 2Co

Theoretical Co loading:

$$\text{Co (wt\%)} = \frac{2\times59}{48\times12+1\quad\times14+4\times16+2\times59}$$

$$\approx 12\ \text{wt\%}$$

c

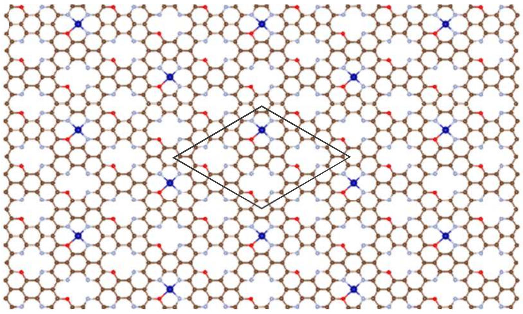

Sample: Co_1/NOC-5

Unit cell: 48C, 12N, 4O, 1Co

Theoretical Co loading:

$$\text{Co (wt\%)} = \frac{1\times59}{48\times12+1\quad\times14+4\times16+1\times59}$$

$$\approx 6\ \text{wt\%}$$

Supplementary Fig. 21 The structure models of various Co_1/NOC-x samples and the corresponding theoretical Co loadings. (a) Co_1/NOC-21, (b) Co_1/NOC-11, and (c) Co_1/NOC-5.

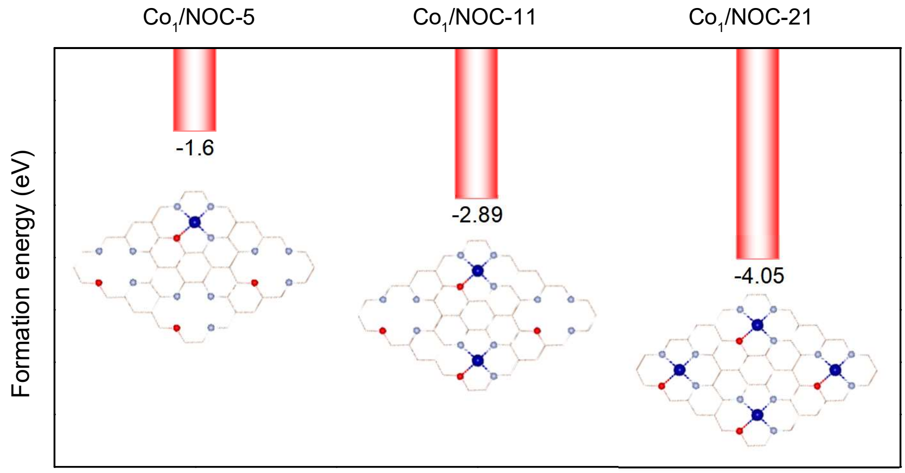

Supplementary Fig. 22 Formation energies of Co_1-N_3O_1 configuration models with varying Co density by DFT calculations. Inset: Optimized structures of 1-Co_1-N_3O_1, 2-Co_1-N_3O_1, and 4-Co_1-N_3O_1 models.

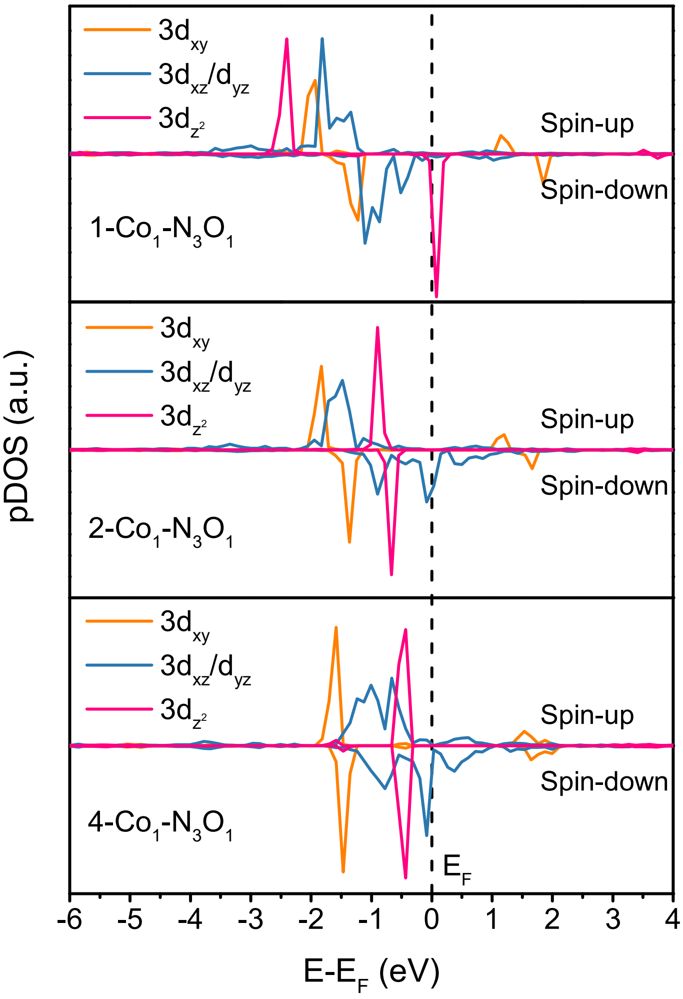

Supplementary Fig. 23 The corresponding spin moment originated from the spin-splitting of Co 3d atom orbitals in x-Co_1-N_3O_1 models.

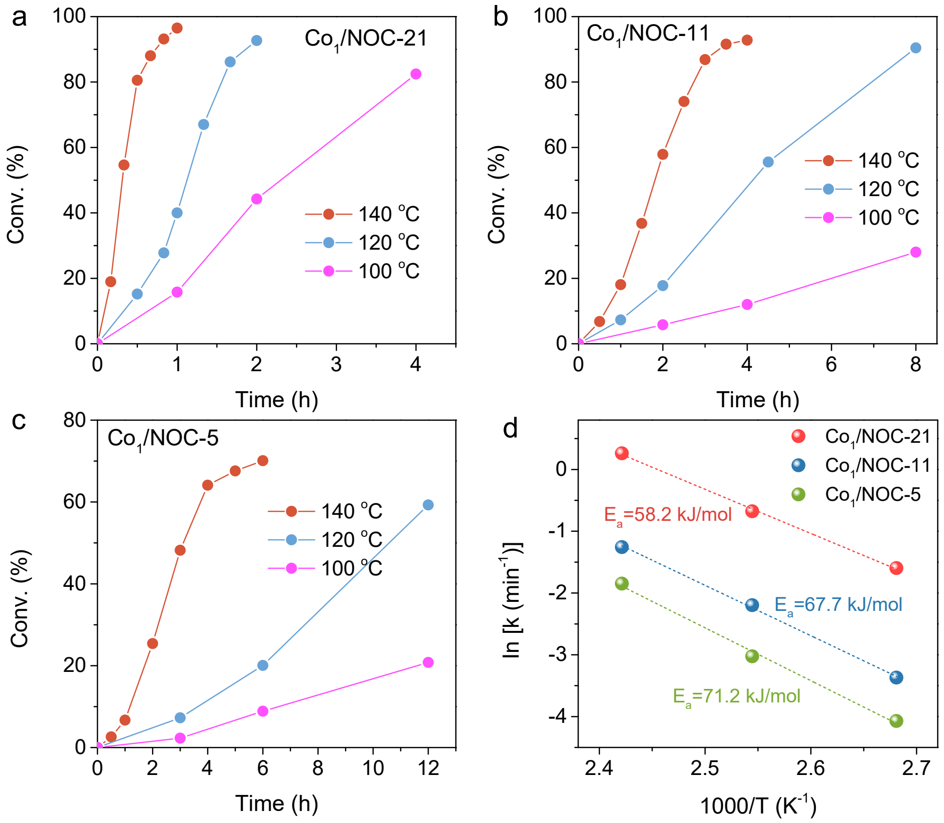

Supplementary Fig. 24 SB conversion against reaction time with various samples of (a) Co_1/NOC-21, (b) Co_1/NOC-11 and (c) Co_1/NOC-5 under different reaction temperatures. (d) Arrhenius plots and corresponding apparent activation energies of the Co_1/NOC-x catalysts.

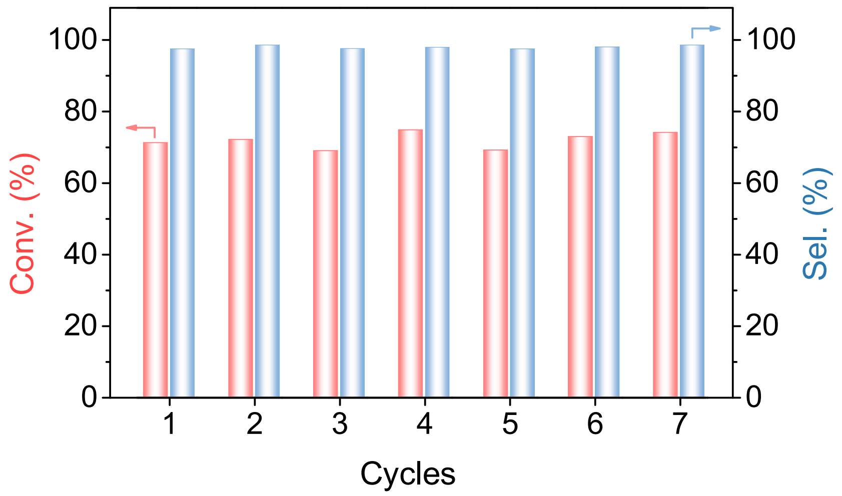

Supplementary Fig. 25 Recycling performances in trans-stilbene epoxidation with Co_1/NOC-21 sample.

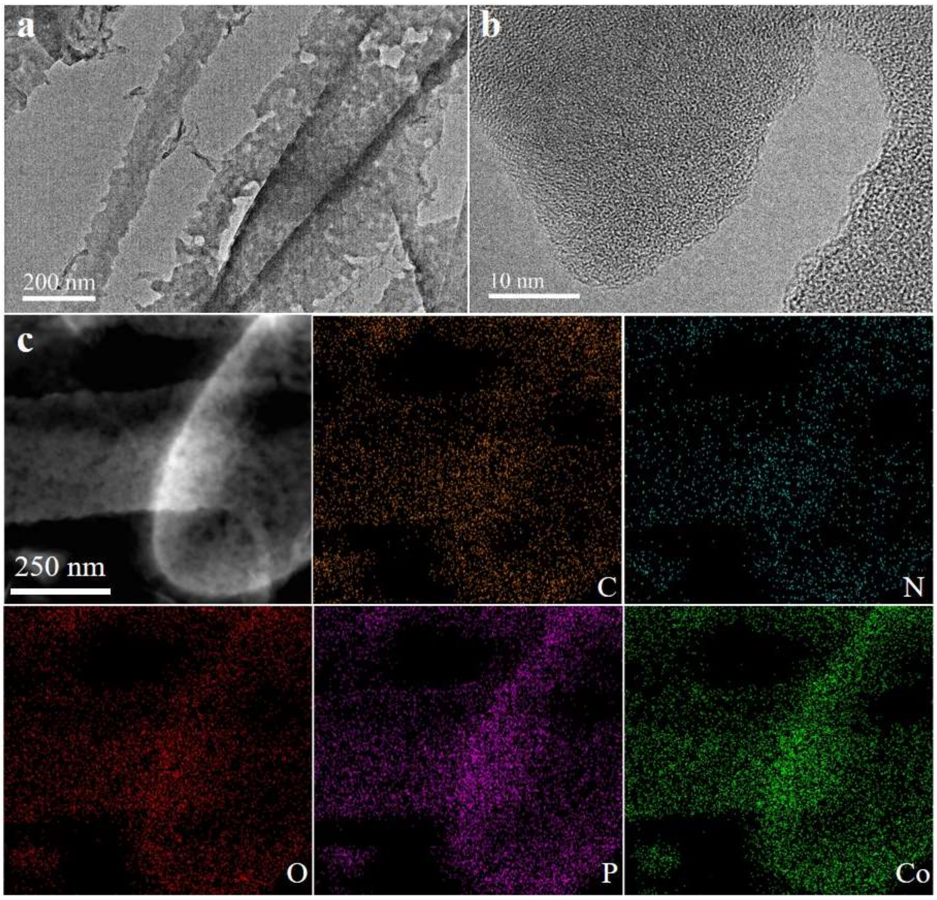

Supplementary Fig. 26 Characterizations of Co_1/NOC-used. (a) TEM and (b) HRTEM images of Co_1/NOC-used. (c) HAADF images and corresponding EDS mapping of Co_1/NOC-used.

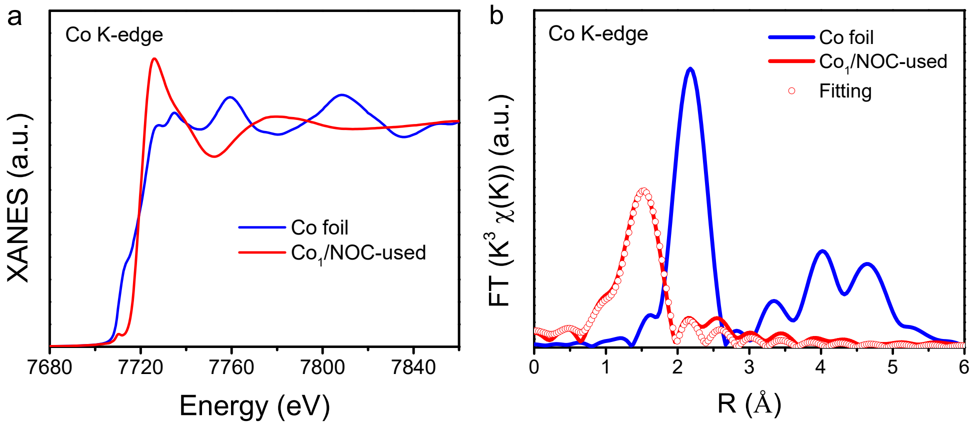

Supplementary Fig. 27 Atomic structural analysis of Co_1/NOC-used. (a) Normalized Co K-edge XANES spectra of Co_1/NOC-used. (b) k^3-weighted Fourier transform spectra of Co_1/NOC-used.

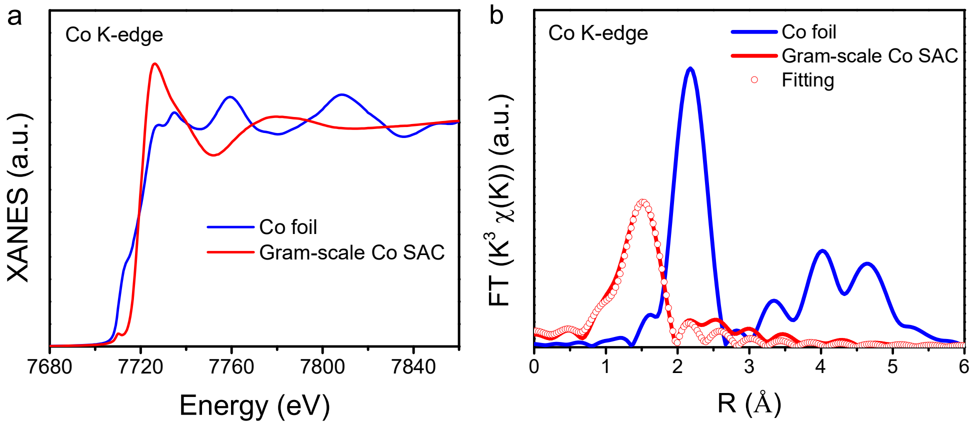

Supplementary Fig. 28 Atomic structural analysis of gram-scale Co SAC. (a) Normalized Co K-edge XANES spectra of gram-scale Co SAC. (b) k^3-weighted Fourier transform spectra of gram-scale Co SAC.

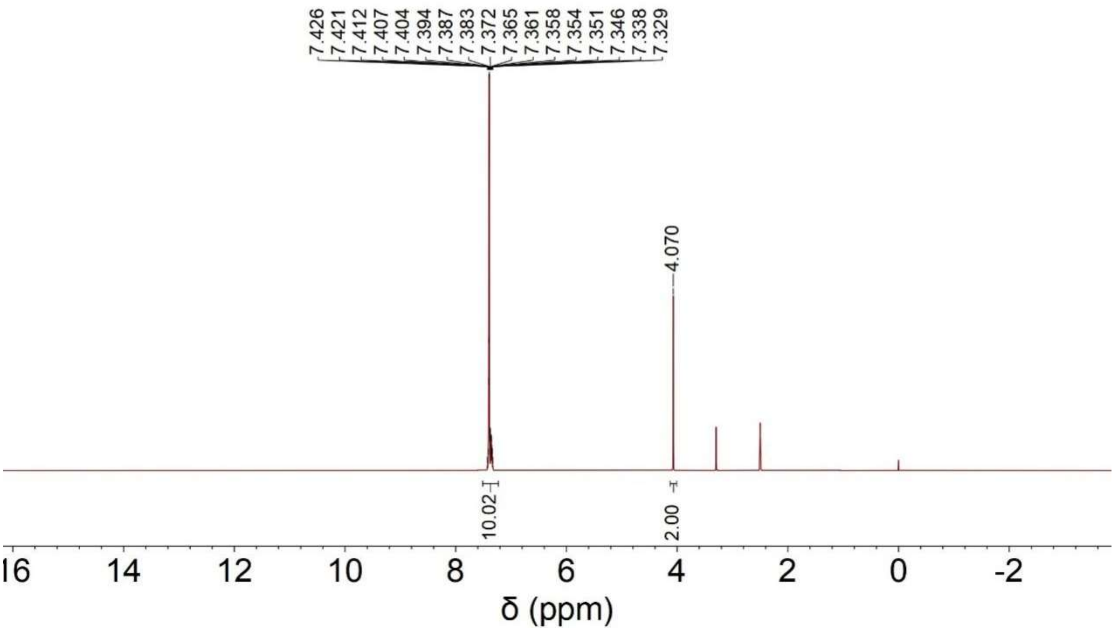

Supplementary Fig. 29 ^{1}H NMR spectra of generated SBO. ^{1}H NMR (400 MHz, DMSO-d_6) δ 7.42 – 7.31 (m, 10H), 4.07 (s, 2H).

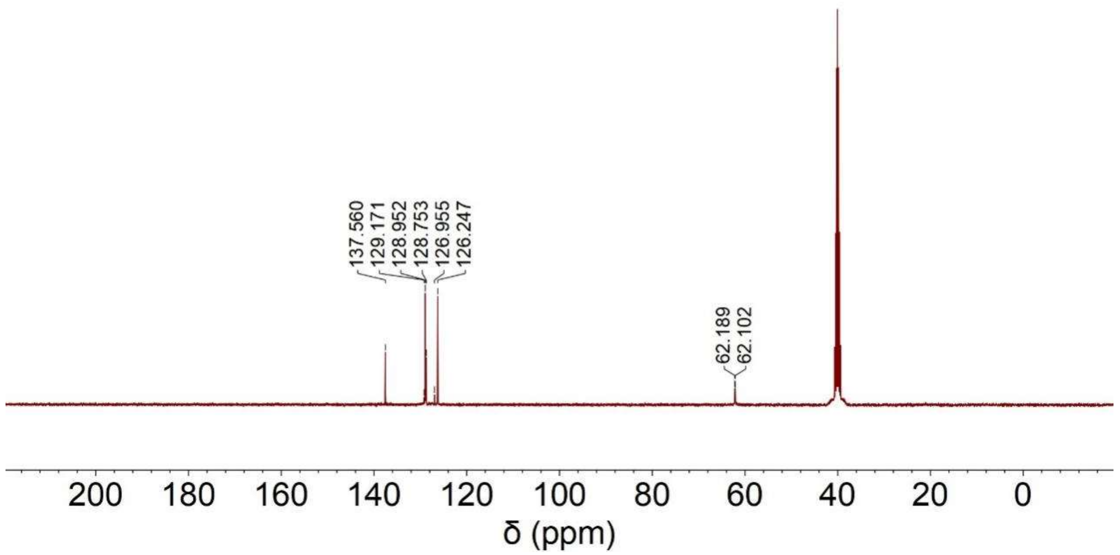

Supplementary Fig. 30 ^{13}C NMR spectra of generated SBO.^{13}C NMR (101 MHz, DMSO-d_6) δ 137.03, 128.42, 128.22, 125.72, 61.61 (d, J = 8.4 Hz).

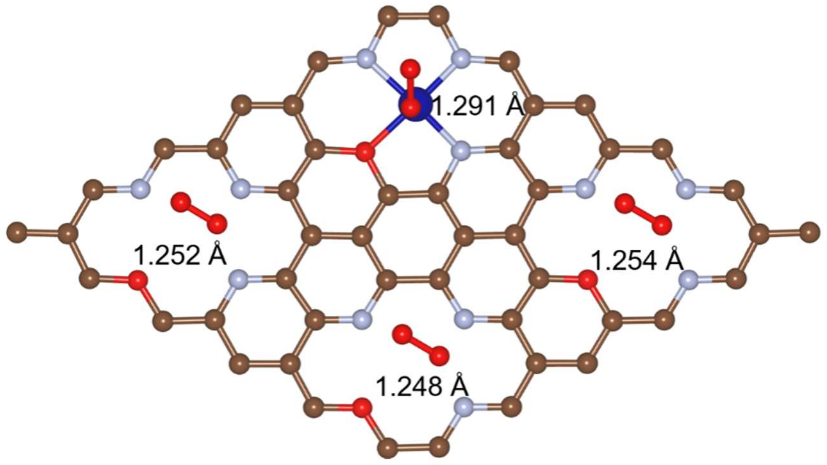

Supplementary Fig. 31 O-O bond after adsorption on Co atom and vacancy sites.

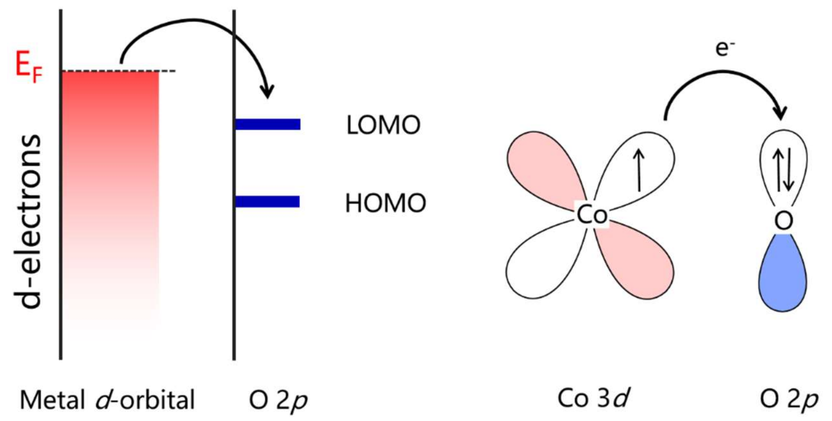

Supplementary Fig. 32 Scheme of the energy levels of Co and O (left) and charge transfer between them (right).

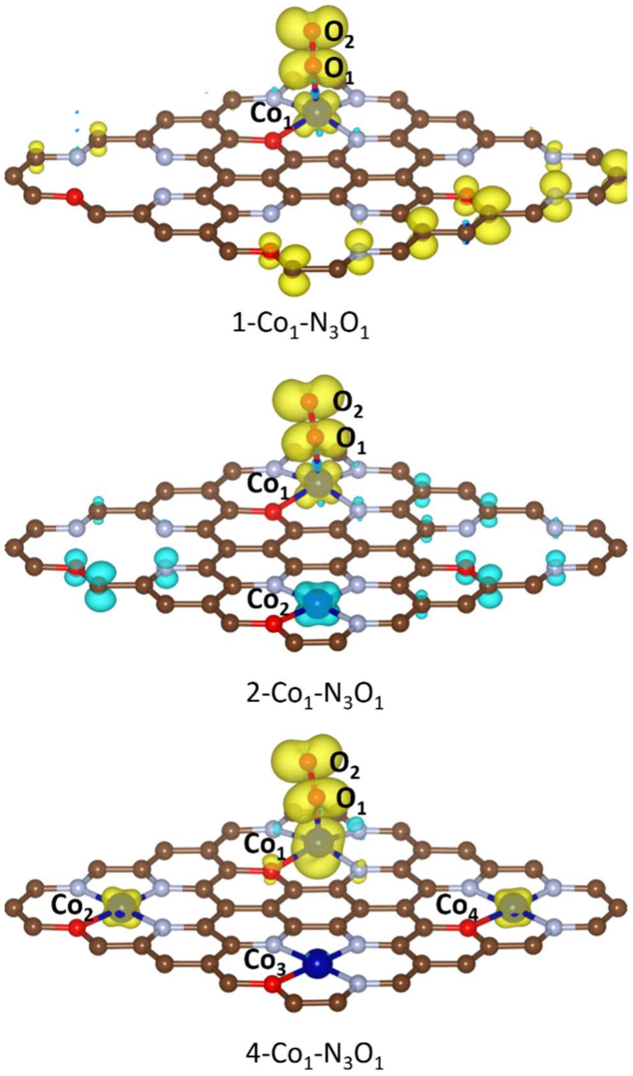

Supplementary Fig. 33 The spin density isosurfaces of various x-Co_1-N_3O_1 models. Isosurfaces: 0.003 e/Å^{-3}.

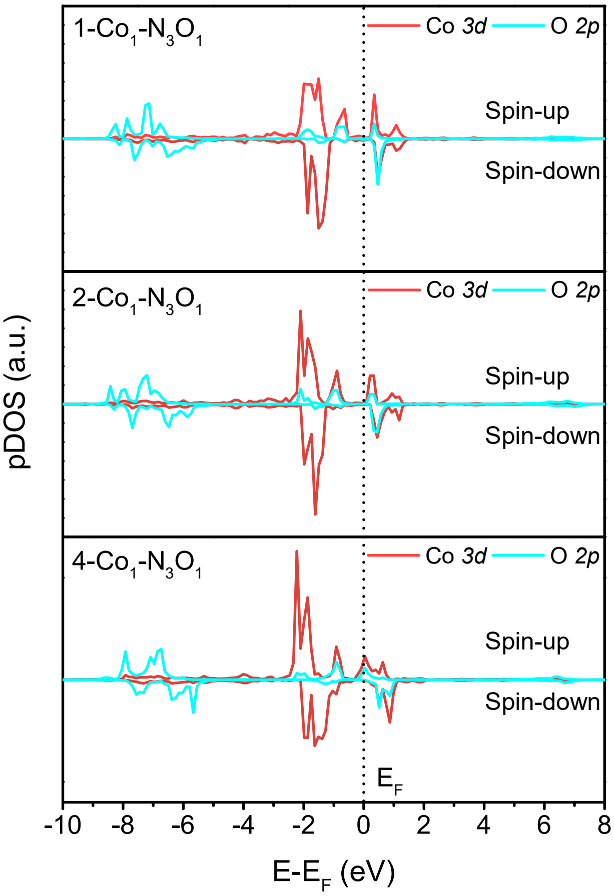

Supplementary Fig. 34 The corresponding spin moment originated from the spin-splitting of Co 3d and O 2p orbitals after O_2 adsorption on various x-Co_1-N_3O_1 models.

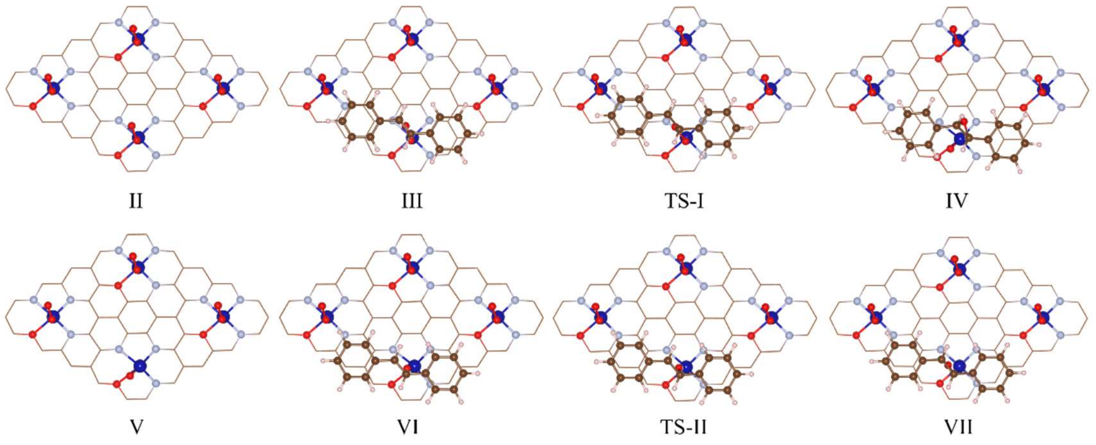

Supplementary Fig. 35 Top view of the intermediate configurations of 4-Co_1-N_3O_1.

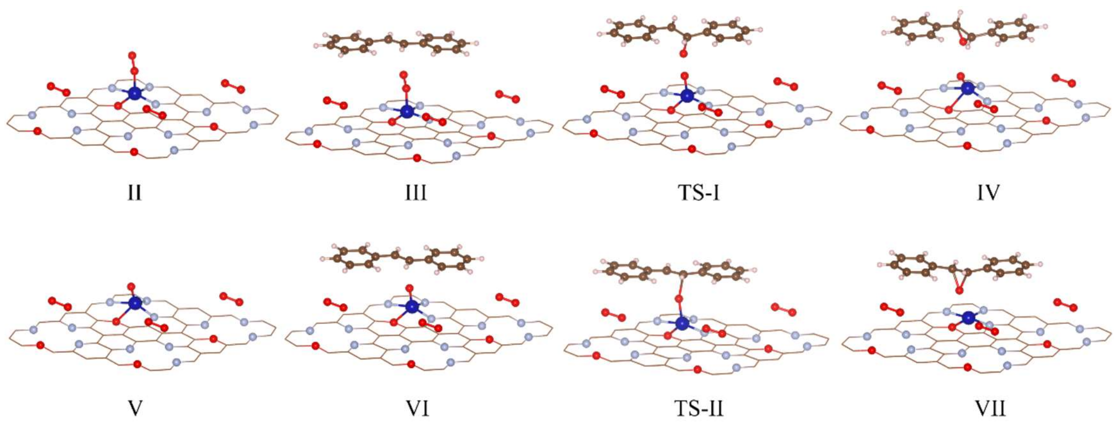

Supplementary Fig. 36 The reaction pathways and the configurations of intermediates over 1-Co_1-N_3O_1.

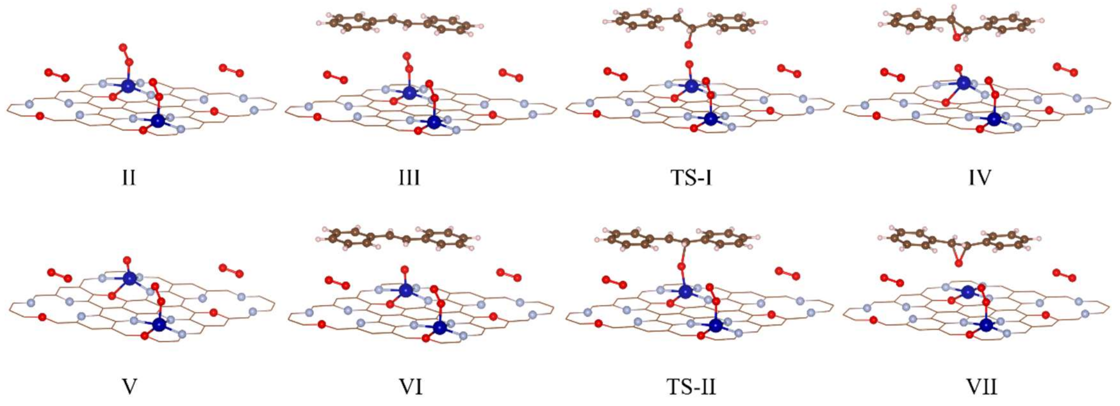

Supplementary Fig. 37 The reaction pathways and the configurations of intermediates over 2-Co_1-N_3O_1.

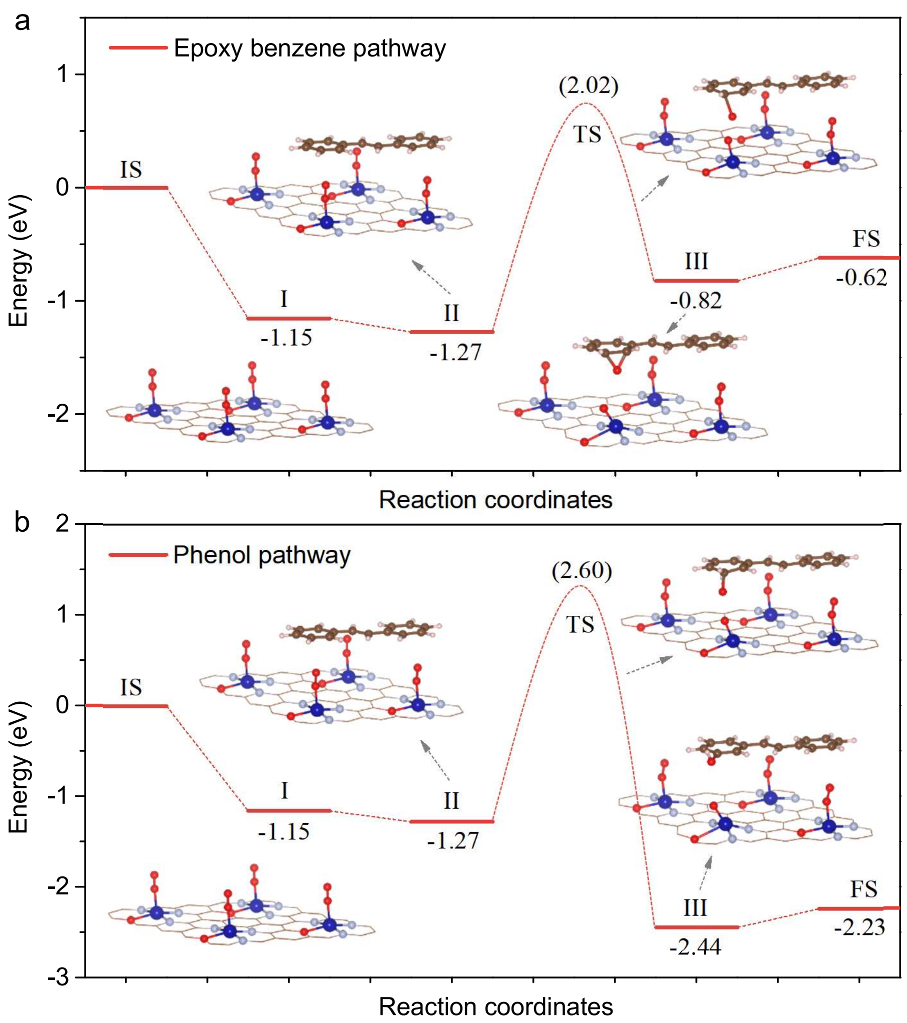

Supplementary Fig. 38 The reaction pathways for formation of (a) epoxy benzene and (b) phenol. Inset: the corresponding configurations of intermediates on 4-Co_1-N_3O_1.

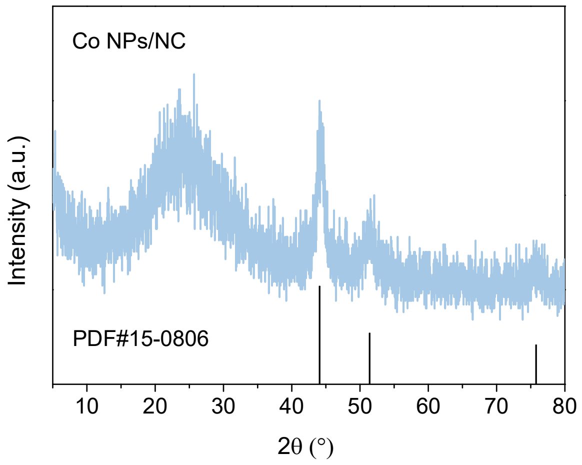

Supplementary Fig. 39 XRD pattern of Co NPs/NC sample.

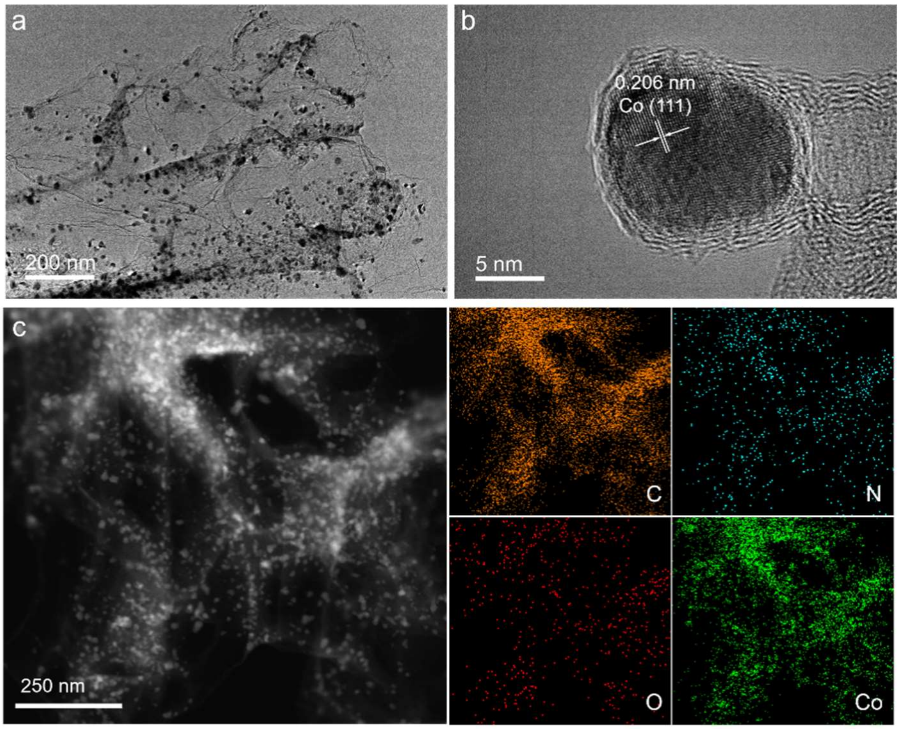

Supplementary Fig. 40 Characterizations of Co NPs/NC sample. (a) TEM and (b) HRTEM images of Co NPs/NC sample. (c) HAADF image and corresponding EDS distribution mapping.

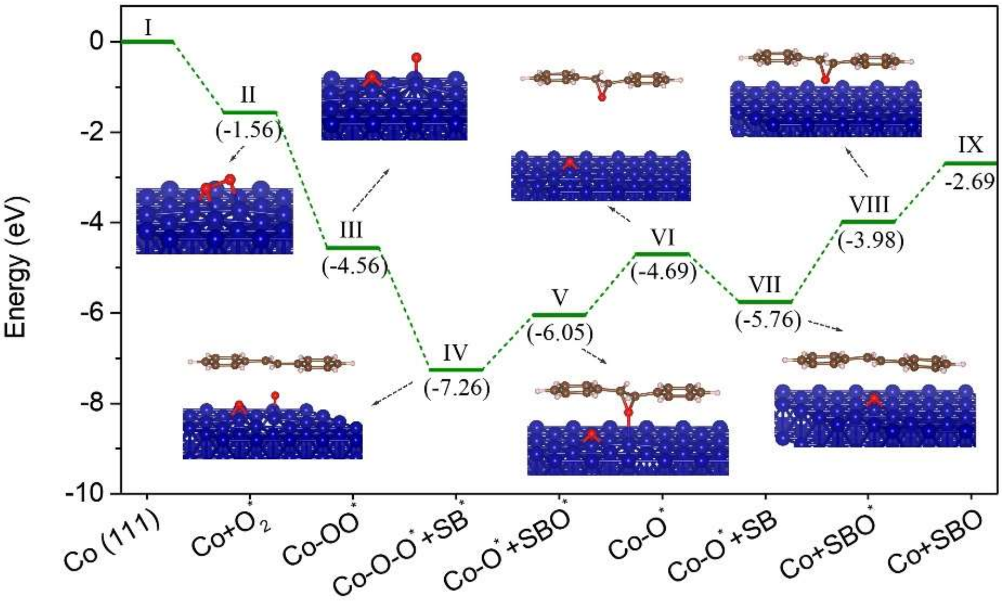

Supplementary Fig. 41 Energy profiles of trans-stilbene epoxidation reaction on Co(111) of Co NPs/NC models. Inset: the configurations of intermediates.

Supplementary Table 1. ICP results of various M_1/NOC samples

Samples	Metal content (wt%)
Fe_1/NOC	21.7
Co_1/NOC	21.2
Ni_1/NOC	16.4
Cu_1/NOC	21.3
Zn_1/NOC	35.5
Ru_1/NOC	17.8
Ir_1/NOC	14.1

Supplementary Table 2. EXAFS fitting parameters for various M-SACs samples

Sample	Shell	N [a]	R (Å) [b]	σ^2 ($Å^2 \cdot 10^{-3}$) [c]	ΔE0 (eV) [d]	*R* factor (%)
Fe_1/NOC	Fe-N/O	3.9	2.04	10.3	3.6	0.9
Ni_1/NOC	Co-N/O	4.4	2.03	8.8	-1.7	0.3
Cu_1/NOC	Ni-N/O	4.2	2.03	14.2	-1.9	1.0
Zn_1/NOC	Zn-N/O	3.8	2.00	6.4	4.3	0.7
Ru_1/NOC	Ru-O/N	3.9	2.03	6.0	-1.3	0.2
Ir_1/NOC	Ir-N/O	3.4	2.03	2.3	13.4	0.5

[a] *N*: coordination numbers; [b] *R*: bond distance; [c] σ^2: Debye-Waller factors; [d] ΔE_0: the inner potential correction. *R* factor: goodness of fit. S_0^2 was set as 0.85 for M-N, which was obtained from the experimental EXAFS fit of reference MPc and MO_x by fixing CN as the known crystallographic value and was fixed to all the samples.

Supplementary Table 3. ICP results of various Co_1/NOC-x samples.

Samples	Co content (wt%)
Co_1/NOC-5	5.4
Co_1/NOC-11	10.9
Co_1/NOC-21	21.2
Co_1/NOC-used	20.6

Supplementary Table 4. EXAFS fitting parameters at the Co K-edge for various samples（S_0^2= 0.769）

Sample	Shell	N^a	R(Å)^b	σ^2(Å^2)^c	ΔE_0(eV)^d	R factor
Co foil	Co-Co	12	2.49	0.0062	6.3	0.0006
CoO	Co-O	6.0	2.06	0.0101	-3.6	0.0004
	Co-Co	12.5	2.99	0.0095		
	Co-O	6.2	3.66	0.0101		
CoPc	Co-N	4.0	1.91	0.0016	6.8	0.0025
Co_1/NOC-5	Co-N/O	4.0	2.04	0.0073	-0.2	0.0003
Co_1/NOC-11	Co-N/O	4.0	2.04	0.0081	-0.4	0.0003
Co_1/NOC-21	Co-N/O	3.8	2.02	0.0084	0.2	0.0005
Co_1/NOC-used	Co-N/O	4.2	2.03	0.0073	-0.1	0.0005

$^a N$: coordination numbers; $^b R$: bond distance; $^c\sigma^2$: Debye-Waller factors; ^{d} ΔE_0: the inner potential correction. R factor: goodness of fit. S_0^2 was set to 0.769, according to the experimental EXAFS fit of Co foil references by fixing CN as the known crystallographic value.

Supplementary Table 5. XPS results of various Co_1/NOC-x samples.

Samples	Elements (at%)				
	C	N	O	P	Co
Co_1/NOC-5	67.83	20.50	7.54	2.88	1.25
Co_1/NOC-11	68.35	19.29	7.22	2.28	2.86
Co_1/NOC-21	66.72	18.01	7.82	2.13	6.22

Supplementary Table 6. Bader charge of various x-Co_1-N_3O_1 models.

Subs	1-Co_1-N_3O_1	2-Co_1-N_3O_1	4-Co_1-N_3O_1
Bader charge (e)	0.8278	0.8175	0.7974
		0.8146	0.7952
			0.7969
			0.7933
Average (e)	0.8278	0.8161	0.7957

Supplementary Table 7. The spin moments of Co atoms in different x-Co_1-N_3O_1 models.

Subs	Co label	1-Co_1-N_3O_1	2-Co_1-N_3O_1	4-Co_1-N_3O_1
spin moment (μ_B)	Co1	0.797	0.438	0.319
	Co2		-0.412	-0.306
	Co3			-0.311
	Co4			0.304
Average (μ_B)	Co	0.797	0.425	0.310

Supplementary Table 8. Substrate scope of alkene epoxidation over the Co_1/NOC-21 catalyst.

Entry	Substrate	Production	Time (h)	Conv. (%)	Sel. (%)
1	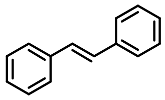	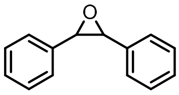	1	96.5	98.1
2	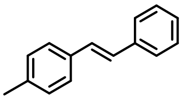	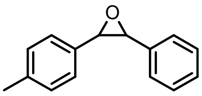	1.5	97.8	99.2
3	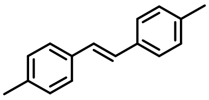	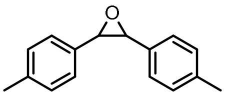	5	91.3	98.5
4	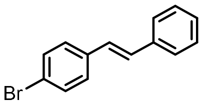	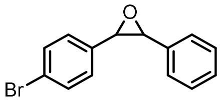	3	99	97.3
5	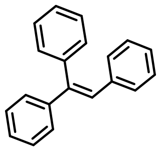	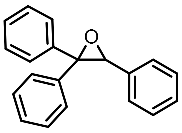	7	>99	>99
6	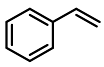	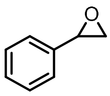	1	76.1	78.9
7	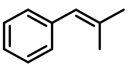	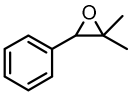	2.5	84.9	97.2

Supplementary Table 9. The spin moments of Co atoms in different O_2-adsorbed x-Co_1-N_3O_1 models.

Subs	Label	1-Co_1-N_3O_1	2-Co_1-N_3O_1	4-Co_1-N_3O_1
spin moment (μ_B)	Co1	0.088	0.159	0.443
	Co2		-0.269	0.149
	Co3			-0.114
	Co4			0.163

Supplementary Table 10. The spin moments of O atoms in different x-Co_1-N_3O_1 models before and after adsorption.

Subs	1-Co_1-N_3O_1	2-Co_1-N_3O_1	4-Co_1-N_3O_1
Before adsorption (μ_B)	1.616		
After adsorption (μ_B)	0.838	0.812	0.770
ΔM (μ_B)	0.778	0.804	0.846

Supplementary Table 11. The sole value of imaginary frequencies of TS-1 and TS-2 on various x-Co_1-N_3O_1 models.

Subs	1-Co_1-N_3O_1	2-Co_1-N_3O_1	4-Co_1-N_3O_1
TS-I (cm^{-1})	427.38	613.61	509.13
TS-II (cm^{-1})	567.23	626.22	400.47

Supplementary Table 12. Free energies of the reaction intermediates with different Co_1-N_3O_1 models.

Step	Model (eV)		
	1-Co_1-N_3O_1	2-Co_1-N_3O_1	4-Co_1-N_3O_1
Co_1	0	0	0
Co_1+O_2*	-1.10	-1.12	-1.15
Co_1+O_2*+SB*	-1.55	-1.48	-1.39
TS-I	-0.76	-0.77	-0.78
Co_1+O*+SBO*	-1.93	-1.98	-1.97
Co_1+O*	-1.50	-1.47	-1.49
Co_1+O*+SB*	-2.07	-2.02	-2.04
TS-II	-1.70	-1.67	-1.86
Co_1+SBO*	-2.93	-2.98	-2.98
Co_1+SBO	-2.62	-2.65	-2.64
